# Transfusion-Related Acute Brain Injury: A Case Report on Reversible Cerebral Vasoconstriction Syndrome

**DOI:** 10.7759/cureus.9077

**Published:** 2020-07-08

**Authors:** Kevin Yeboah, Jan Bittar, Mohammad Almajali, Momina Soudagar Turkey, Joanna Ramiro

**Affiliations:** 1 Neurology, Saint Louis University School of Medicine, St. Louis, USA; 2 Neurocritical Care, The Ohio State University Wexner Medical Center, Columbus, USA

**Keywords:** rcvs, transfusion reaction, pres, vasospasm, thunderclap headache, seizure, dysautoregulation, rbcs

## Abstract

Reversible cerebral vasoconstriction syndrome (RCVS) manifests with a thunderclap headache and reversible vascular abnormalities. Red blood cell transfusions have not been well identified as a risk factor for RCVS. We report a rare case of acute brain injury resulting from RCVS after a packed red blood cell (PRBC) transfusion. A 49-year-old female with a history of menorrhagia initially presented with generalized weakness. She was found to have a hemoglobin (Hgb) of 1.7 g/dL in the setting of a fundal fibroid for which she received five units of PRBCs. Post transfusion, she complained of several days of thunderclap headache and later returned with new-onset seizures. She was admitted to the neurocritical care unit for the treatment of status epilepticus. Metabolic, infectious and toxic work-up were unremarkable except for an elevated lactate. MRI of the brain with contrast showed extensive bilateral hemispheric and cerebellar white matter T2-weighted fluid-attenuated inversion recovery (T2/FLAIR) hyperintensities with areas of enhancement. A diagnostic cerebral angiogram was performed to evaluate for a vascular etiology and revealed focal segmental stenoses in bilateral A1 segments of the anterior cerebral arteries and in branches of the bilateral middle cerebral arteries. These findings were suggestive of RCVS. Clinicians should have a high degree of suspicion for RCVS in patients presenting with neurological manifestations, such as thunderclap headache or seizures after recent transfusion. The window for injury may be longer than that seen in other organs, such as in transfusion-related acute lung injury (TRALI).

## Introduction

Reversible cerebral vasoconstriction syndrome (RCVS) manifests with a thunderclap headache and transient multifocal segmental cerebral artery vasoconstriction [1]. This clinical-angiographic syndrome occurs between the ages of 20 and 50 years with a higher prevalence in women [1,2]. Although RCVS is commonly reversible, it is associated with several neurological complications, including seizure, ischemic and hemorrhagic strokes [3]. The pathophysiology of RCVS is thought to be related to impaired cerebral vascular tone. Common risk factors for RCVS include the use of nasal decongestants, antidepressants and substances of abuse, such as amphetamines, cocaine and ecstasy [2]. Red blood cell (RBC) transfusions are the mainstay in treatment of severe blood loss; however, they have not been well identified as a risk factor for RCVS [4]. We report a rare case of acute brain injury resulting from RCVS after transfusion of RBCs. 

## Case presentation

A 49-year-old female with a history of menorrhagia initially presented to an outside hospital with generalized weakness. She was found to have a hemoglobin (Hgb) of 1.7 g/dL. She received five units of packed red blood cells (pRBCs) and her Hgb improved to 8.5 g/dL. A pelvic and transvaginal ultrasound demonstrated a fundal fibroid, and she was started on medroxyprogesterone for perimenopausal hormonal imbalance. 

Seven days later, she presented to our facility with new-onset seizures. Her family also reported that she had been complaining of severe throbbing headache for several days prior to admission. The seizures were generalized, tonic-clonic with right gaze deviation. She was loaded with intravenous (IV) levetiracetam after adequate dosages of IV lorazepam per status epilepticus protocol. The seizures continued and IV fosphenytoin was administered resulting in seizure cessation. On examination, she was afebrile, hypertensive to 174/100 and non-verbal with impaired vision. She had increased flexor tone in the bilateral upper extremities with extensor tone in the lower extremities. 

**Figure 1:**
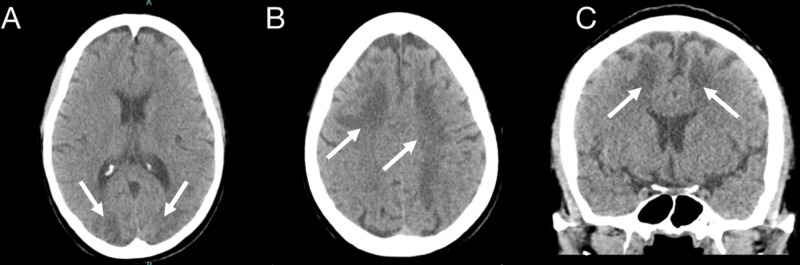
CT of the head without intravenous contrast: axial (A, B) and coronal (D) views Hypodense areas in the subcortical regions of the bilateral cerebral hemispheres involving the frontal, parietal and occipital lobes (arrows)

Metabolic work-up revealed a high anion gap metabolic acidosis (HAGMA) with a lactic acid of 10.3 mmol/L and a pH of 7.13. Infectious and toxic work-up were unremarkable. A CT of the head demonstrated hypodense areas in the subcortical regions of the bilateral cerebral hemispheres (Figure 1). 

**Figure 2:**
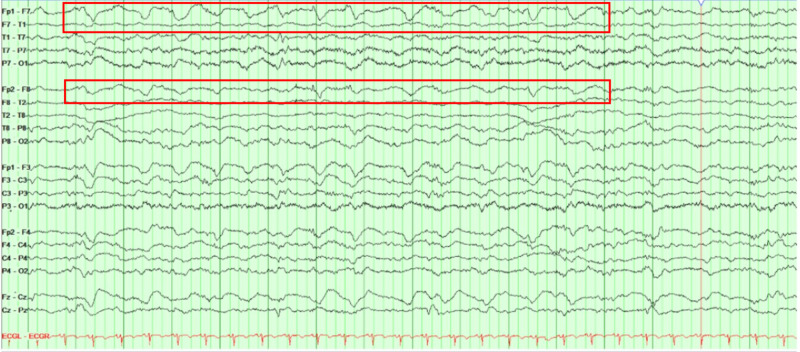
A longitudinal bipolar montage (double banana) continuous electroencephalogram (cEEG) Generalized background slowing with bilateral frontal intermittent rhythmic delta activity (FIRDA) of 1 to 1.5 Hz left > right

A continuous electroencephalogram (cEEG) was obtained to rule out subclinical seizures and showed generalized background slowing with bifrontal intermittent rhythmic delta activity (FIRDA) (Figure 2).

**Figure 3:**
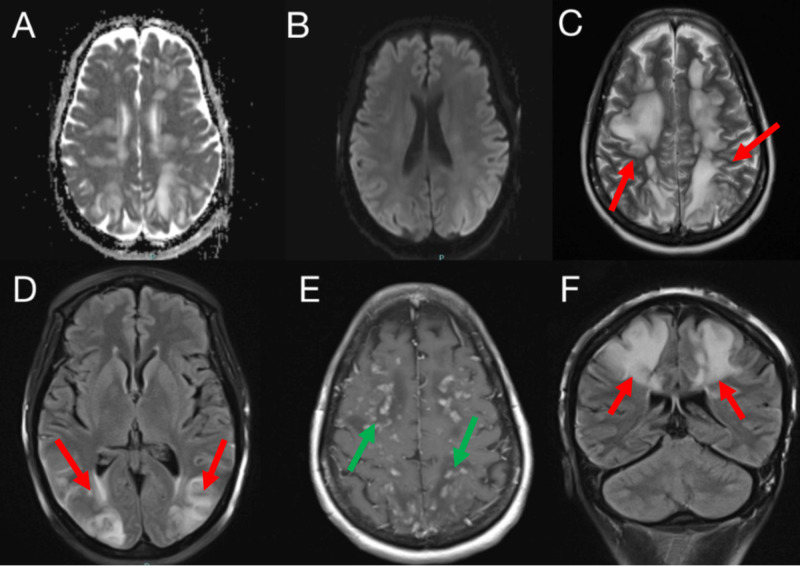
MRI of the brain with and without intravenous contrast: A (axial ADC), B (axial DWI), C (axial T2), D (axial T2 FLAIR), E (axial T1 with contrast) and F (coronal T2 FLAIR) Extensive confluent patchy areas of T2 and FLAIR hyperintensities involving the subcortical white matter of the bilateral frontal, parietal and occipital lobes (red arrows). Patchy foci of enhancement within the bilateral cerebral hemispheres (green arrows). No associated diffusion restriction ADC, apparent diffusion coefficient; DWI, diffusion-weighted imaging; FLAIR, fluid-attenuated inversion recovery

A follow-up MRI of the brain with contrast showed extensive bilateral hemispheric and cerebellar white matter T2-weighted fluid-attenuated inversion recovery (T2/FLAIR) hyperintensities with areas of enhancement (Figure 3). There was no evidence of large artery occlusions or venous sinus thrombosis on the magnetic resonance arteriography or venography. 

**Figure 4:**
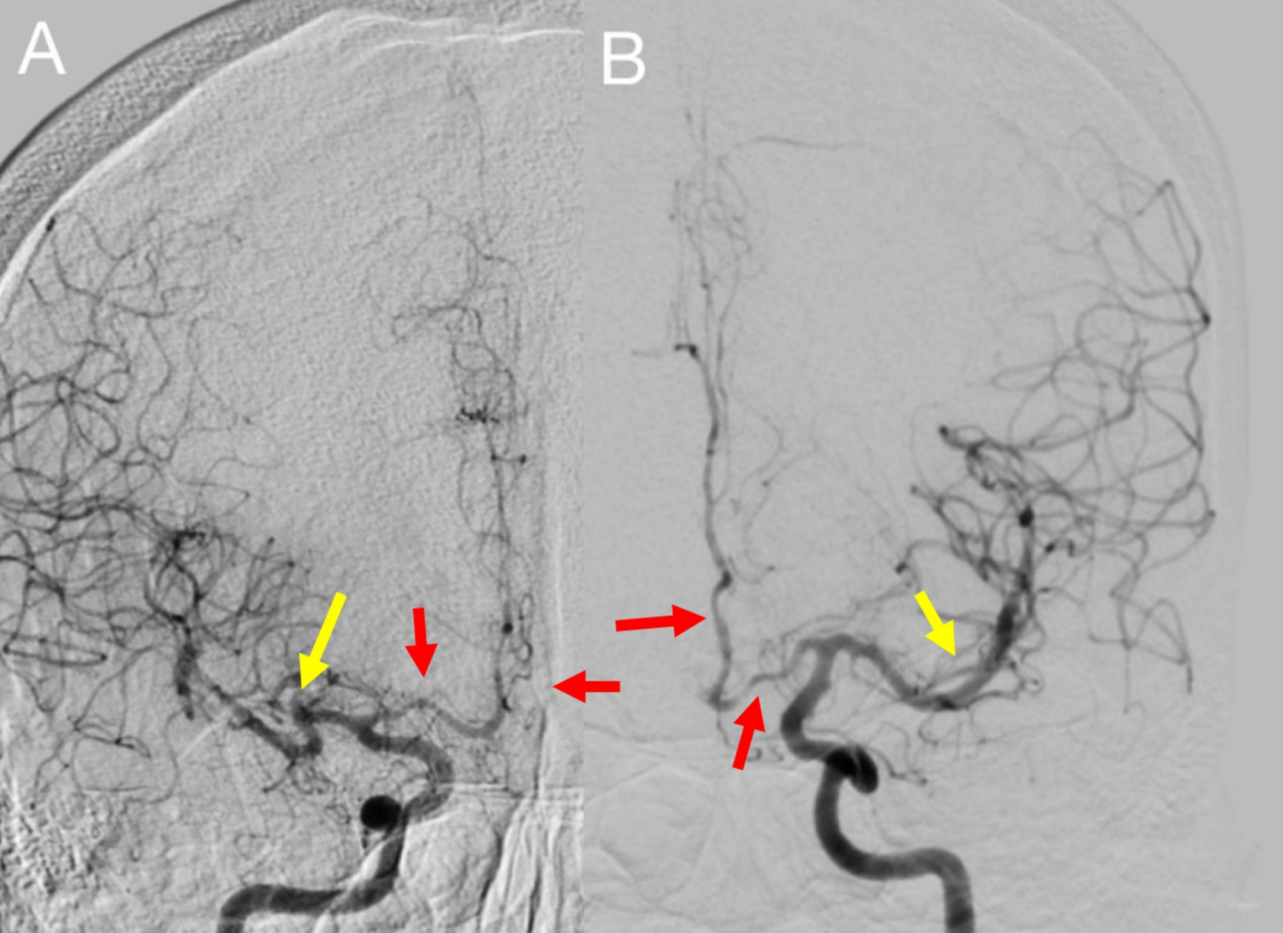
Digital subtraction angiogram of the bilateral internal carotid arteries (ICA). A: right ICA, B: left ICA Focal areas of stenosis in the bilateral proximal A1 and A2 segments of the anterior cerebral arteries (red arrows) and bilateral superior M2 branches of the middle cerebral arteries (yellow arrows)

She was started on a nicardipine drip for elevated blood pressure along with broad-spectrum antibiotics and acyclovir for meningoencephalitis coverage. She was admitted to the neurocritical care unit for continuous monitoring. A lumbar puncture was performed, and cerebral spinal fluid (CSF) studies revealed an RBC count of 5,150/µl with negative xanthochromia and normal levels of glucose and protein, suggestive of a traumatic spinal tap. Subsequently, meningoencephalitis coverage was discontinued. The patient remained encephalopathic and unresponsive. A diagnostic cerebral angiogram was performed to evaluate for a vascular etiology and revealed focal segmental stenoses in bilateral A1 segments of the anterior cerebral arteries and in branches of the bilateral middle cerebral arteries (Figure 4). These findings were suggestive of RCVS. 

Ten days after admission, her mental status gradually improved and she became responsive to questions and commands. She had no further seizures and reported an improvement in her headache. Her blood pressure was optimized, and she was transferred out of the neurocritical care unit and later discharged to an acute rehabilitation center. 

## Discussion

We report a rare case of RCVS after a PRBC transfusion. The mechanism for induction of RCVS in the setting of a blood transfusion is not well defined. Trends in the literature suggest that risk factors for RCVS after a blood transfusion include chronicity of anemia and an increase in Hgb levels greater than 5 g/dL [5]. 

A thunderclap headache is the hallmark of RCVS [6]. Establishment of recurrent headaches is of diagnostic importance. Liang et al. found headaches to be the most common presenting symptom in RCVS [7]. Recurrent headaches in the first few days of symptom onset have been demonstrated in 82%-100% of patients, many experiencing three to four headaches [1]. A severe throbbing headache followed by new-onset seizures was the presenting feature in our case. Seizures can be a presenting symptom and have been reported in 1%-17% of RCVS cases [6]. RCVS can occur spontaneously; however, precipitating factors commonly associated with this condition include the use of vasoactive drugs, such as serotonergic and adrenergic agents, the postpartum period, unruptured cerebral aneurysms, carotid-cervical or vertebral artery dissections and posterior reversible encephalopathy syndrome (PRES) [6]. RCVS and PRES share many clinic-radiological features suggestive of similar pathophysiology [6]. In PRES, dysautoregulation from a sudden elevation in blood pressure leads to cerebral hyperperfusion and impairment of the blood-brain barrier causing vasogenic edema [8]. Similarly, in RCVS, a transient failure of cerebral vascular tone causes multifocal arterial constriction and dilation [3]. 

The brain is not an organ commonly associated with blood transfusion reactions. Furthermore, most research has been conducted on transfusion-related acute lung injury (TRALI). The pathophysiology of TRALI is not fully understood. Two mechanisms believed to be associated with TRALI include leuko-agglutination, which occurs after transfusion of blood products containing leukocyte antibodies, and a lipid-mediated lung injury that occurs secondary to accumulation of lipids from prolonged blood storage [9]. A combination of autoregulatory responses to changes in blood volume and oxygenation may be pathogenic in blood transfusion associated RCVS. As with cerebral misery perfusion seen in patients with chronic carotid occlusions, a compensatory vasodilation may occur in response to impaired oxygenation [10]. Rapid correction of Hgb during transfusion can result in loss of vasodilation [7]. The increase in hematocrit and viscosity with administration of blood products may induce an acute cerebral vascular endothelial injury and subsequent vasospasm [11]. The rapid transfusion of blood products in our case, increasing the Hgb from 1.7 to 8.5 g/dL, may have predisposed the cerebral vascular system to angiopathy. 

Kothari et al. reported a case of a patient with iron-deficiency anemia whose Hgb was also increased by 5 g/dL and subsequently developed RCVS after two weeks [11]. This was consistent with our patient who presented one week after having received a transfusion, and this suggests that the time to onset of symptoms can be delayed. The quantity and duration of the time over which blood is transfused may underlie this delay [7]. A slow centripetal involvement of the vasculature, with small arteries being affected first, may also account for this latency [7]. 

Approximately 8% to 38% of patients with RCVS will have reversible cerebral edema [6]. In our case, the areas for focal segmental stenosis seen on digital subtraction angiogram corresponded with the extensive parenchymal distribution seen on MRI. The resolution of edema usually occurs within one month, which precedes the reversal of vasoconstriction [6]. A repeat MRI 10 days after admission demonstrated improvement in previously seen hyperintensities in this specific patient. 

## Conclusions

It is important to consider the brain as an organ at risk of injury during blood transfusion. Clinicians should have a high degree of suspicion for RCVS in patients presenting with neurological manifestations, such as thunderclap headache or seizures after recent transfusion. The window for injury may be longer than that seen in other organs, such as in TRALI. 
